# Cutaneous and Mucosal Manifestations Associated with Celiac Disease

**DOI:** 10.3390/nu10070800

**Published:** 2018-06-21

**Authors:** Luis Rodrigo, Valia Beteta-Gorriti, Nuria Alvarez, Celia Gómez de Castro, Alvaro de Dios, Laura Palacios, Jorge Santos-Juanes

**Affiliations:** 1 Gastroenterology Unit, Hospital Universitario Central de Asturias (HUCA), Avda. de Roma s/n, 33011 Oviedo, Asturias, Spain; nuriaalvarezh@gmail.com; 2 Dermatology Unit, Hospital Universitario Central de Asturias (HUCA), Avda. de Roma s/n, 33011 Oviedo, Asturias, Spain; valita_bg@hotmail.com (V.B.-G.); celiagomez_88@hotmail.com (C.G.d.C.); aldedivel@gmail.com (A.d.D.); llaurinapalacios@hotmail.com (L.P.); 3 Facultad de Medicina, Universidad de Oviedo, 33003 Oviedo, Asturias, Spain

**Keywords:** celiac disease, dermatitis herpetiformis, urticaria, atopic dermatitis, psoriasis, recurrent aphtous ulceration, rosacea, alopecia areata, cutaneous vasculitis, gluten-free diet

## Abstract

Celiac disease (CD) is an immune-mediated, gluten-induced enteropathy that affects predisposed individuals of all ages. Many patients with CD do not report gastrointestinal symptoms making it difficult to reach an early diagnosis. On the other hand, CD is related to a wide spectrum of extra-intestinal manifestations, with dermatitis herpetiformis (DH) being the best characterized. These associated conditions may be the clue to reaching the diagnosis of CD. Over the last few years, there have been multiple reports of the association between CD and several cutaneous manifestations that may improve with a gluten-free diet (GFD). The presence of some of these skin diseases, even in the absence of gastrointestinal symptoms, should give rise to an appropriate screening method for CD. The aim of this paper is to describe the different cutaneous manifestations that have been associated with CD and the possible mechanisms involved.

## 1. Introduction

Celiac disease (CD) is a chronic autoimmune systemic disease associated with an enteropathy triggered by gluten intake which affects genetically predisposed individuals of both sexes and can develop at any age. Gluten and its major protein fractions, gliadin and glutenin, are present in wheat, rye, barley, oats, related species and hybrids, and processed foods [1]. Almost all patients with CD present the human leukocyte antigen (HLA) DQ2 (>90%) or HLA DQ8 (5–10%); nevertheless, up to 40% of people in the Americas, Europe, and Southeast Asia also carry these alleles, indicating that these genes are necessary but not sufficient for CD development [2]. The findings of inflammatory changes in intestinal biopsies, ranging from lymphocytic enteritis to various degrees of villous atrophy, are the gold standard for CD diagnosis, even in the presence of a negative serology for CD. IgA anti-tissue transglutaminases are the most sensitive and cost-effective antibodies for the diagnosis of CD, although deamidated gliadin peptide IgG antibodies might be useful in seronegative patients with innate IgA deficiency. A life-long gluten-free diet (GFD) is mandatory, achieving clinical and histological recovery in most patients [1].

In past decades, CD was considered to be an uncommon disease affecting mainly children and limited to individuals of European ancestry. Currently, we know that this disorder may be detected at any age and is regarded as one of the most common chronic diseases encountered worldwide with a prevalence of about 1–2% [3]. The mean age of adult CD diagnosis is 45 years, although up to 20% of patients are diagnosed at the age of 60 years or above. CD is probably an under-diagnosed entity in adulthood partly because many patients in this age group lack the classical symptoms, such as diarrhea or signs of malabsorption. In fact, in most adult patients, gastrointestinal symptoms are subtle or even absent, and clinical suspicion arises from extra-intestinal manifestations (non-classic or atypical CD), such as anemia, cutaneous disorders, neurological disease, osteoporosis, and abnormal liver function tests [2,4]. We emphasize the importance of considering non-typical symptoms to diagnose adult CD and actively searching extra-intestinal associated manifestations in order to start an early GFD and prevent the onset of long-term complications.

CD patients are more frequently affected by other immune-mediated disorders (ID) compared to the general population, as reported in previous studies, mainly thyroid and skin diseases and type 1 diabetes mellitus [5,6]. This observation may be partially explained by a possible spread of the adaptive immune response, initially triggered in the gastrointestinal tract, to other tissues [4,6]. Hashimoto’s thyroiditis is the most frequently associated ID, followed by several skin disorders, such as psoriasis, atopic dermatitis (AD), vitiligo, systemic lupus erythematosus (SLE), alopecia areata (AA), and oral lichen planus (OLP) [6]. Interestingly, 60% of CD patients with associated thyroid disease that develop a third ID are skin related. These data suggest a relationship among the immunological systems of the thyroid, skin, and small bowel, which seem to be more susceptible to developing aberrant immunological responses against auto-antigens. [6,7,8,9,10,11,12].

Cutaneous manifestations associated with CD, other than dermatitis herpetiformis (DH), are poorly known. It is currently recognized that DH is an undoubted extra-intestinal manifestation of CD. In addition, there is growing evidence that supports the link between CD and several skin disorders. In 2006, Humbert et al. proposed a classification of skin diseases associated with CD, dividing them into four categories: autoimmune, allergic, inflammatory, and miscellaneous (Table 1) [9,10,11,12,13,14,15,16,17,18,19]. Recently, Bonciolini et al. described 17 patients affected by non-celiac gluten sensitivity with skin manifestations similar to eczema, psoriasis, and DH who did not show a specific histological pattern [20]. The only common findings in most of these patients were severe itching, the presence of C3 at the dermo–epidermal junction and rapid resolution after adopting a GFD. The authors emphasized the importance of close collaboration between gastroenterologists and dermatologists due to the multiple associations between gastrointestinal and skin disorders. In the present paper, we aim to describe the multiple skin disorders associated with CD and the possible mechanisms involved.

## 2. Immunopathogenesis of Skin and Oral Lesions Associated with CD

The immune responses in CD are very wide. A probable explanation lies in the presence of an increase in intestinal permeability in both groups of patients, in relation to the direct toxic effect of gliadin on the surface of the intestinal epithelium [21,22]. This enables the passage of gluten peptides and other related peptides to the bloodstream, provoking the appearance of different inflammatory or autoimmune processes that may affect any organ or tissue, which can be the result of aberrant immune responses [21,23,24]. As Hadjivassiliou stated more than 15 years ago, “that gluten sensitivity is regarded as principally a disease of the small bowel is a historical misconception.” [22,25].

In the submucosa of the small intestine, starting with the the action of tissue transglutaminase type 2 which unfolds gluten, a cascade of events occurs, causing a Th1 response that stimulates B lymphocytes that release IgE and other immunoglobulins [26] which play a important roles in the appearance of urticaria and AD, and a stimulation of Th2 mediated by T-lymphocytes which produces the release of pro-inflammatory cytokines, such as TNF-α and interferon gamma (IFNγ), among others [27], and that play important roles in several types of immune-mediated dermatitis, such as psoriasis. In addition, these immunological responses can also cause the production of circulating immunocomplexes due to antigen–antibody interactions which predominate in vasculitic lesions.

## 3. Dermatitis Herpetiformis

DH is a common extra-intestinal manifestation of CD. A special review article on this disease was recently published in the May 2018 special issue in Nutrients [28]. In summary, DH presents with itchy vesicles and papules, mainly on the elbows, knees, and buttocks. Overt gastrointestinal symptoms are rare. Although in duodenal biopsies, up to 75% of patients with DH with varying degrees of villous atrophy are observed, predominantly mild to moderate, it should be taking into account that in the remaining 25%, only inflammatory changes of changes of lymphocytic enteritis are observed, in the absence of villous atrophy. A diagnosis of DH is easily confirmed by biopsies showing pathognomonic granular immunoglobulin A (IgA) deposits in the papillary dermis by direct immunofluorescence. A valid hypothesis for the immune pathogenesis of DH is that it starts from latent or manifested CD in the gut and evolves into an immune complex deposition of high avidity IgA epidermal transglutaminase (TG3) antibodies, together with the TG3 enzyme, in the papillary dermis. The DH to CD prevalence ratio is 1:8 in Finland and the United Kingdom (UK). The annual DH incidence rate, currently 2.7 per 100,000 in Finland and 0.8 per 100,000 in the UK, is decreasing, whereas the reverse is true for CD. The long-term prognosis of DH patients on a GFD is excellent, with the mortality rate being even lower than for the general population [28].

## 4. Urticaria

**Figure 1:**
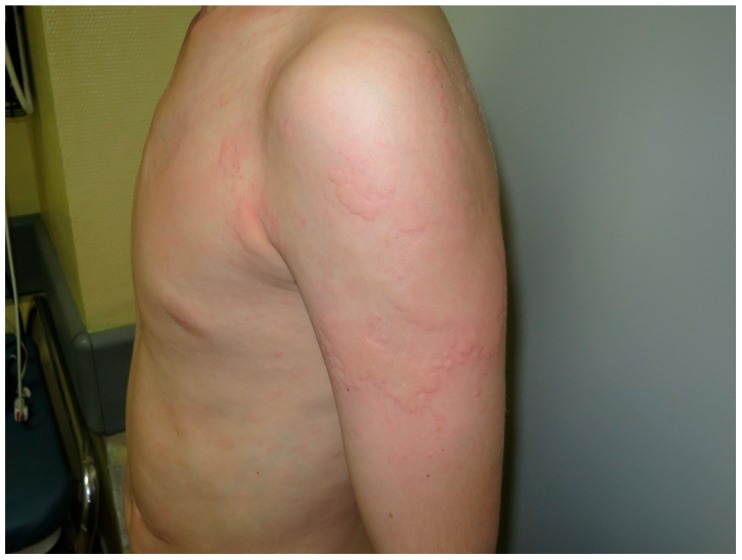
Urticaria. Pale to red, well-demarcated, transient swellings, involving the dermis, mainly at the thorax and the left arm.

Urticaria is characterized by the onset of wheals, angioedema, or both (Figure 1) [29]. Urticaria is a common disorder, occurring in 15–25% of individuals at some point in life [29,30]. Chronic urticaria (CU) (duration ≥6 weeks) is seen in about 0.5–1% of the general population [31,32]. CU is associated with a substantial decrease in quality of life [31]. The etiopathogenesis of CU is thought to be associated with autoimmune mechanisms [33,34,35,36]. CU has been shown to have a genetic association with the human leukocyte antigen HLA-DQ8 alleles [37]. Interestingly, HLADQ8 has an association with CD [37,38].

In 1987, Hauteke et al. first described the association between CD and chronic urticaria [39], although the relationship between these two diseases is not fully clear. Recently, Kolkhir et al. stated that chronic spontaneous urticaria is strongly linked with various autoimmune diseases, including Hashimoto’s thyroiditis, pernicious anemia, vitiligo, diabetes mellitus type 1, Grave’s disease, rheumatoid arthritis, and CD [40]. In a large population study, 453 patients with CD and no previous diagnosis of urticaria developed urticaria, and 79 of these 453 patients had chronic urticaria [12]. The corresponding hazard ratios were 1.51 for any urticaria (95%CI = 1.36–1.68) and 1.92 for chronic urticaria (95%CI = 1.48–2.48). These data support an increased prevalence of urticaria and chronic urticaria in patients with CD [12].

In some cases of CU, the adoption of a GFD has proven its effectiveness in controlling skin flares [41,42], further sustaining that CU may be a cutaneous manifestation of CD and not only a fortuitous association [11].

## 5. Atopic Dermatitis

**Figure 2:**
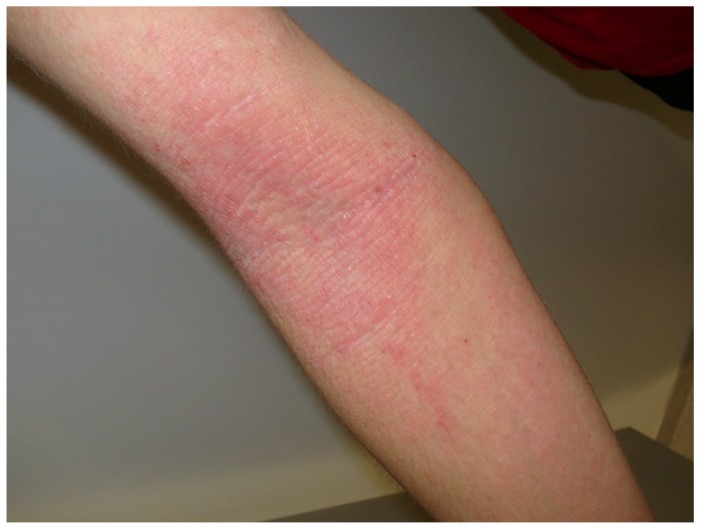
Atopic dermatitis. Excoriated bilateral erythematous scaling papules and plaques on the right flexor elbow surface.

AD is a chronic inflammatory skin disease that is associated with a heterogeneous group of symptoms and signs. The cutaneous signs of AD include erythema, lichenification, scaling, and prurigo nodules (Figure 2). The symptoms of AD include cutaneous itch and pain [43], sleep disturbance and fatigue [44,45], and mental health symptoms [46,47,48]. All of these manifestations contribute to diminish the quality of life, limiting the ability to perform activities of daily life and causing psychosocial distress and stigma [49]. AD affects 40 million individuals worldwide [50], and its prevalence is still increasing. Notably, AD appears to be more prevalent among children under five years of age, and its prevalence decreases with advancing age [51]. The onset of AD occurs primarily in childhood and is thought to precede allergic disorders mediated by immunoglobulin E (IgE) sensitization to environmental antigens, namely AD, asthma, and allergic rhino-conjunctivitis, the so-called atopic triade [52,53,54,55]. Though extensive recent studies have shed light on the understanding of AD, the exact pathogenesis of the disease is still unknown. The complex interaction between genetics, environmental factors, microbiota, skin barrier deficiency, immunological derangement, and possibly autoimmunity contributes to the development of the disease [56,57,58,59].

AD has also been linked with CD. Ress et al. analyzed the prevalence of CD in 351 children with AD compared with a general pediatric population and showed a four-fold greater risk of developing CD in patients with AD (OR, 4.18; 95% CI, 1.12–15.64) [60]. This study also emphasizes the need to evaluate the cost-effectiveness of screening patients with AD for CD in time to prevent long-term complications. Moreover, Ciacci et al. conducted a case control study involving 4114 adult patients, with and without CD, and observed that AD was three-fold more frequent in patients with CD and two-fold more frequent in their relatives than in their spouses (OR, 3.17; 95% CI, 1.02–9.82) [13].

## 6. Psoriasis

**Figure 3:**
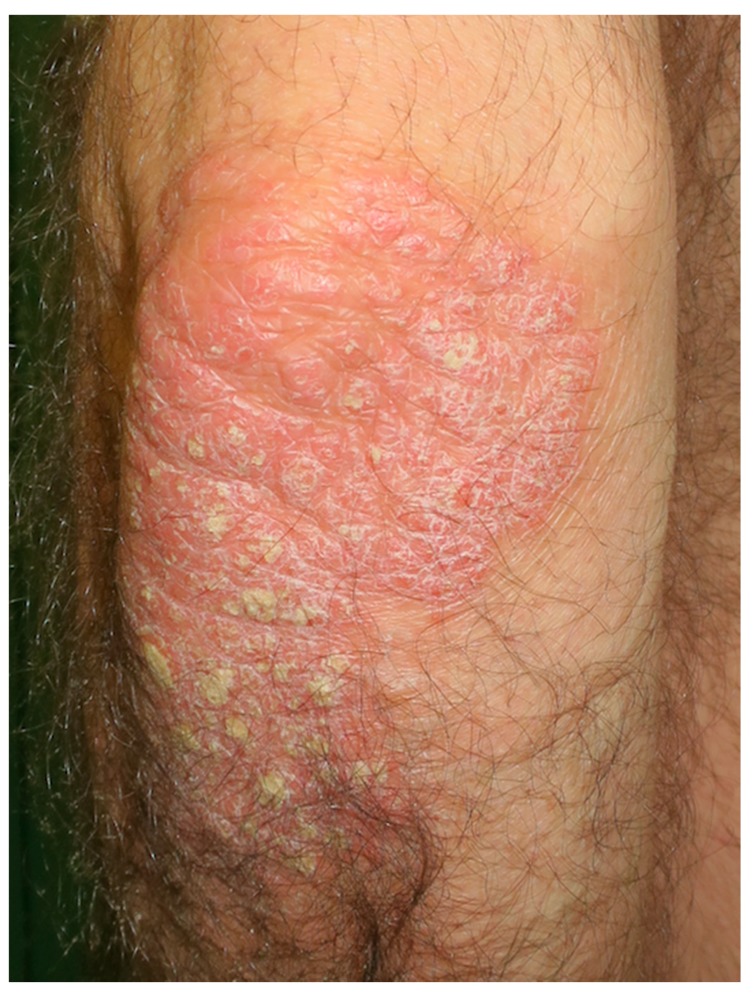
Extense plaque of psoriasis at the left elbow extensor side.

**Figure 4:**
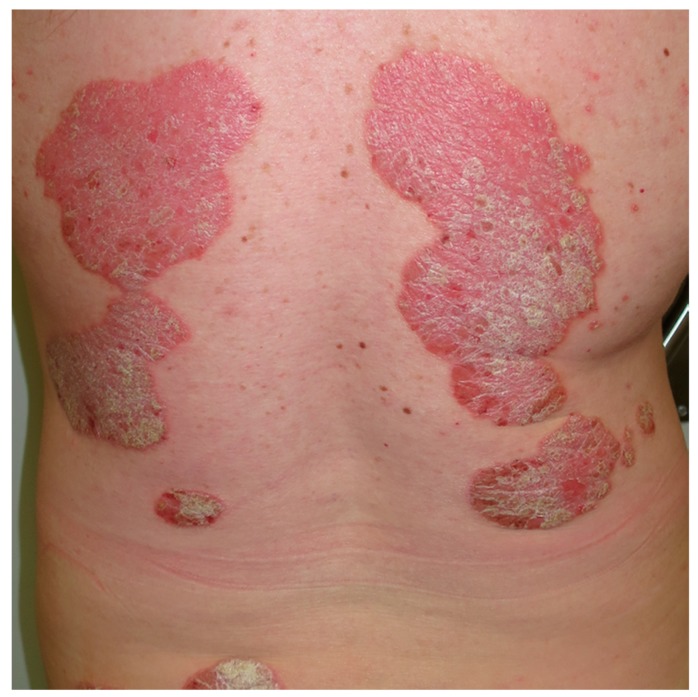
Psoriasis. Well demarcated, erythematous, scaly plaques that are relatively symmetrical on the back.

Psoriasis is an autoimmune chronic inflammatory skin disease with an estimated prevalence of 2–4% in the adult population [61,62]. It affects over 7.5 million people in the United States and approximately 125 million people worldwide [54]. Psoriasis is considered to be a multifactorial disease, in which the genetic background interacts with environmental factors to define an individual’s risk [62,63,64]. The classical clinical manifestations of psoriasis consist of the presence of red, infiltrated plaques, covered with a coarse, silvery scaling (Figure 3 and Figure 4). Predilection sites include the elbows and knees, scalp, and periumbilical and lumbar regions, although any anatomical site might be affected [65]. The clinical course of psoriasis is marked by frequent relapses with fluctuating rates [62].

Psoriasis is known to be associated with an increased risk of several comorbidities, including inflammatory arthritis, metabolic syndrome, and atherosclerotic disease [63]. The association between psoriasis and CD has been of recent interest, but its first recognition was in 1971 by Marks and Shuster [66]. They described, for the first time, a “psoriatic enteropathy” in a small group of patients with severe psoriasis. For many years, the relationship between psoriasis and CD has remained controversial since the few available data were inconclusive. A recent meta-analysis demonstrated a significantly higher risk of CD among patients with psoriasis compared with participants without psoriasis with the OR of 3.09 (95%CI = 1.92–4.97) [16]. Furthermore, seven studies have reported a positive association between psoriasis and CD markers [66,67,68,69,70,71,72]. In contrast, other studies did not find evidence of an association between psoriasis and CD markers. However, these studies were of smaller size and some did not employ control groups [73,74,75,76,77]. To resume the evidence for CD antibody positivity in psoriasis, Bhatia et al. performed a meta-analysis of nine studies that reported the frequency of IgA anti-gliadin antibody (IgA AGA) positivity in psoriasis cases and controls [17]. They found a statistically significant higher relative risk of having positive IgA AGA in patients with psoriasis compared to controls (OR = 2.36, 95% CI 1.15–4.83). Other two studies suggested that levels of CD antibodies correlate with psoriasis or psoriatic arthritis severity [78,79]. The use of AGA determination for the diagnosis of CD has low sensitivity, and its use in clinical practice is being abandoned, being replaced by other types of antibodies, such as anti-transglutaminase and deaminated peptides of gliadin [6,21].

The pathophysiologic mechanisms behind the increased risk of CD among patients with psoriasis are not known, but there are different hypotheses that try to explain them [16,80]. The association between CD and several autoimmune diseases, such as type I diabetes mellitus and autoimmune thyroid disease, is well-documented [81,82]. It is believed that shared genes (at-risk HLA haplotypes) might be responsible for this association. The shared genes might play similar roles in the association between psoriasis and CD. Genetic-wide association studies of these two conditions identified genetic susceptibility loci at eight genes that regulate innate and adaptive immune responses: *TNFAIP3*, *RUNX3*, *ELMO1*, *ZMIZ1*, *ETS1*, *SH2B3*, *SOCS1* and *UBE2L3* [80,83,84,85]. Another possible explanation is that the increased proliferation rate of keratinocytes found in patients with psoriasis is known to produce an excessive amount of interleukin (IL)-1 and IL-18, the essential signals for the induction of Th1 response. Interestingly, mucosal inflammation in patients with CD is also caused by the activation of Th1 in response to dietary gluten [86]. Therefore, it is possible that these ILs might predispose patients to CD. On the other hand, it is possible that intestinal barrier dysfunction associated with undiagnosed or untreated CD may allow the increased passage of immune triggers resulting in an increased risk of autoimmune diseases, including psoriasis [86,87]. Finally, CD-related malabsorption may affect psoriasis by causing a vitamin D deficiency status [9,88]. It is well known that low levels of vitamin D predispose individuals to psoriasis and that exposure to sunlight and topical administration of vitamin D analogues improves psoriatic lesions, probably due to its immunoregulatory properties [88].

Although available data regarding the coexistence of CD and psoriasis are still inconclusive, there is a considerable amount of evidence that suggests that psoriatic patients with concomitant CD may benefit from a GFD [17,21,80,89]. Furthermore, the prevalence of the anti-gliadin IgA antibody is significant higher among patients with psoriasis without a diagnosis of gluten-related disorders. For this reason, anti-gliadin IgA testing can identify patients who are likely to benefit from GFDs [90].

To summarize, the relatively frequent coexistence of CD and psoriasis justifies monitoring of patients with either condition for clinical evidence of the other. This is especially important in the case of psoriasis, as it could be the only manifestation of an undiagnosed CD, even in the absence of obvious digestive symptoms. It is advisable to perform the entire protocol to actively search for CD, including duodenal biopsies, even when serological markers are negative. In the case of negative CD findings, performing a trial with a GFD is currently the recognized diagnostic method [23].

## 7. Aphthous Stomatitis

Numerous authors have described a wide variety of oral cavity disorders in patients with CD, and some of these manifestations may be considered diagnostic clues in silent, atypical forms of CD [91].

**Figure 5:**
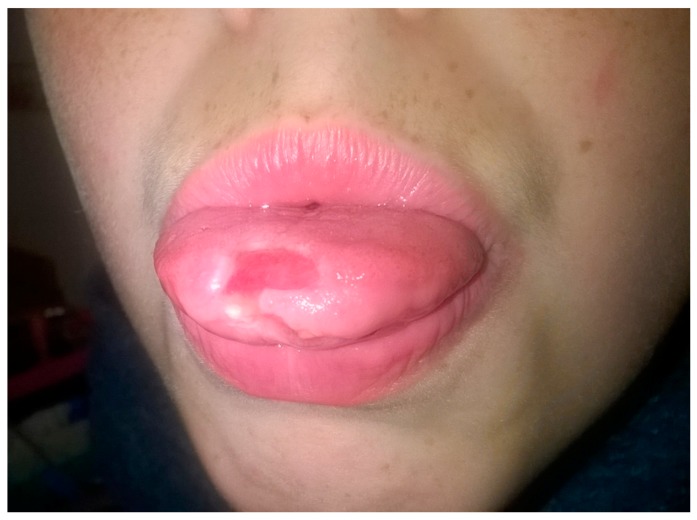
Aphthous lesion on the tip of the tongue, on the upper side.

Recurrent aphthous stomatitis (RAS) is a common clinical condition that produces painful ulcerations in the oral cavity. RAS is characterized by multiple recurrent small, round, or ovoid ulcers with circumscribed margins, erythematous haloes, and yellow or gray floors, typically first presenting in childhood or adolescence [92,93] (Figure 5). RAS has been recognized for many years as a symptom of CD (CD) [93,94,95,96]. A recent meta-analysis showed that celiac patients have greater frequency of RAS (OR = 3.79, 95%CI = 2.67–5.39). When only the children were considered, the OR was 4.31 (95%CI = 3.03–6.13), while in the adults, the OR of only one study was 47.90 (95%CI 6.29–364.57) [18]. RAS patients should be considered at-risk subjects, even in the absence of any gastrointestinal symptoms and should therefore undergo a diagnostic procedure for CD [97]. RAS may also be present in patients with DH [98]. A study reported non-specific mucosal ulcers in up to 40% of patients with DH [99]. The etiopathology of RAS is obscure; it is not known whether RAS lesions are directly influenced by the gluten sensitivity disorder, or if these are related to hematinic deficiency with low levels of serum iron, folic acid, and vitamin B12 or trace element deficiencies due to malabsorption in patients with untreated CD [96].

## 8. Rosacea

**Figure 6:**
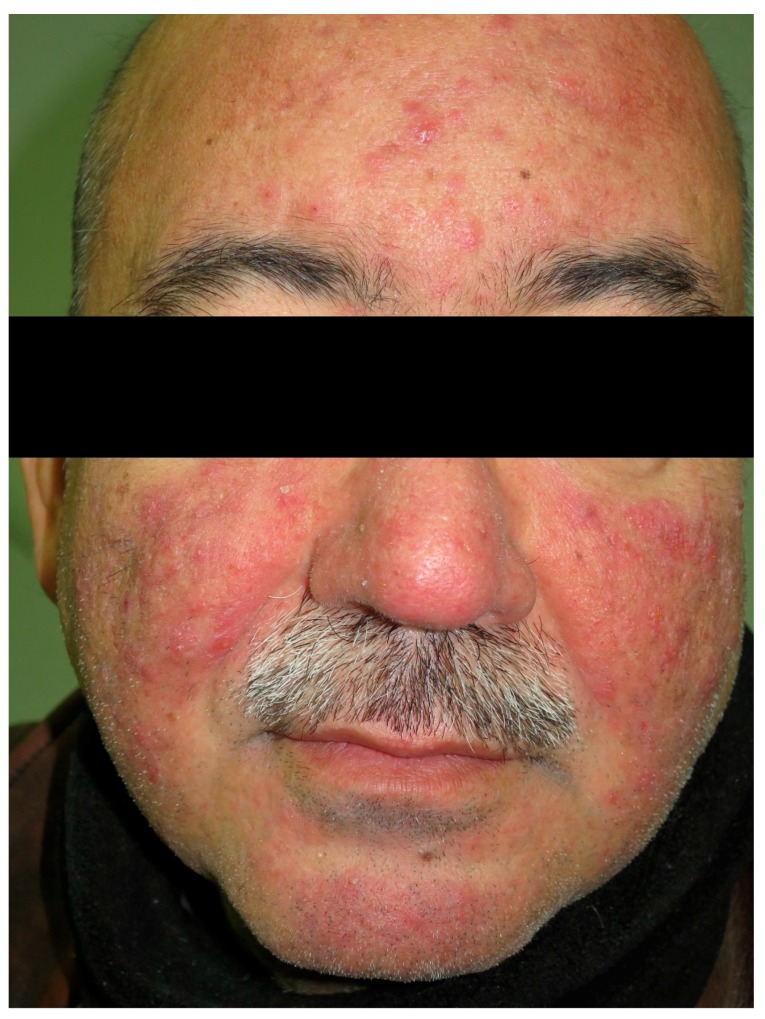
Rosacea. Papule-pustular lesions on the face.

Rosacea is an inflammatory skin condition characterized primarily by persistent or recurrent episodes of centrofacial erythema, with women being more affected than men [100] (Figure 6). The pathophysiology is not completely understood, but dysregulation of the immune system as well as changes in the nervous and vascular systems have been identified [101]. Rosacea can seriously affect a patient’s quality of life, and this should prompt clinicians to diagnose it early and start treatment [100]. Rosacea shares genetic risk loci with autoimmune diseases, such as type 1 diabetes mellitus and CD [102]. One study showed that women with rosacea had a significantly increased risk of CD (OR = 2.03, 95%CI 1.35–3.07) [103]. In a nationwide cohort study, the prevalence of CD was higher among patients with rosacea when compared to control subjects (HR = 1.46, 95%CI = 1.11–1.93) [19]. In this study, rosacea was associated with an increased prevalence of Crohn’s disease, ulcerative colitis, irritable bowel disease, small intestinal bacterial overgrowth, and *Helicobacter pylori* infection.

## 9. Alopecia Areata

**Figure 7:**
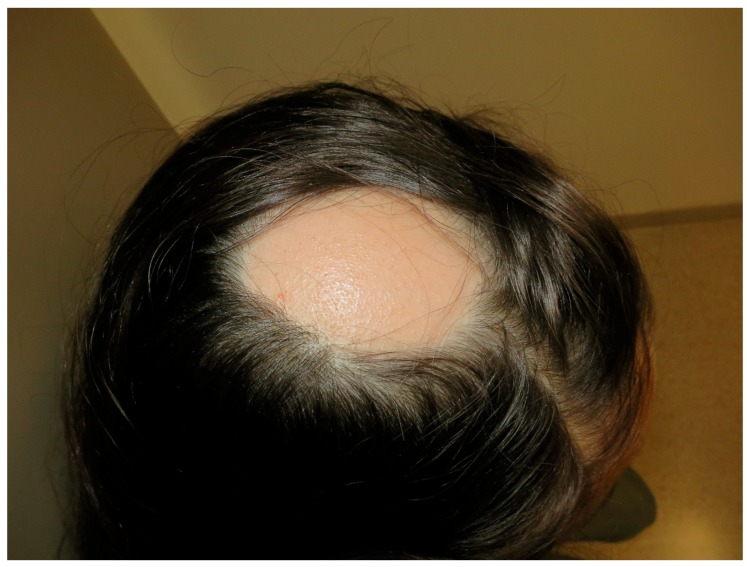
Alopecia areata. Patchy head hair loss.

AA is an autoimmune disease that presents as a non-scarring type of hair loss. AA affects both sexes equally, affects patients of all ages, and is found in approximately 2% of the general population [104]. Clinical presentation of AA is very heterogeneous, ranging from small and well-circumscribed patches of hair loss to a complete absence of body and scalp hair (Figure 7). Exclamation point hairs, dystrophic hairs, and yellow dots are features of AA that can be identified with trichoscopy. Nail abnormalities, such as pitting, brittleness, or striations are seen in 10% to 20% of patients. The main factors affecting prognosis include age at onset and disease extent; younger age at initial presentation and severity at onset are the most important prognostic indicators [105]. The etiology of AA remains unclear, though it is believed to result from a loss of immune privilege in the hair follicle, autoimmune-mediated hair follicle destruction, and the upregulation of inflammatory pathways [105].

AA is associated with other autoimmune disorders, such as Addison’s disease, autoimmune thyroiditis, atrophic gastritis, systemic lupus erythematous, rheumatoid arthritis, myasthenia gravis, and vitiligo [106]. In 1995, Corazza et al. described, for the first time, the association between AA and CD [107]. Since then, there have been other reports of this association. The estimated prevalence rate of CD in patients with AA is from 1:85 to 1:116 [108,109], similar to that found in the general population, so it could be considered to be a random association. However, due to the fact that alopecia improves and even disappears with a GFD, its presence should indicate the possible existence of an undiagnosed CD [11,108,109,110]. In addition, the prevalence of anti-gliadin antibodies in patients with AA was 18:100 in a study conducted in 2011, occurring more often in severe variants of AA, in particular, alopecia universalis [109]. An active search for CD using serological screening tests has been recommended to diagnose the numerous cases of subclinical CD [9], but a recent study stated that the biological tests to search for CD do not bring enough information and proof to disclose gluten intolerance in AA patients [111].

The positive effects of a GFD on the pattern of autoimmune conditions associated with CD, such as AA, have been attributed to the normalization of the immune response [109]. Although remission and recurrence may be observed during the clinical course of AA, many patients on a GFD have shown complete regrowth of the scalp and other body hair and no further recurrence of AA at follow-up [112].

## 10. Cutaneous Vasculitis

**Figure 8:**
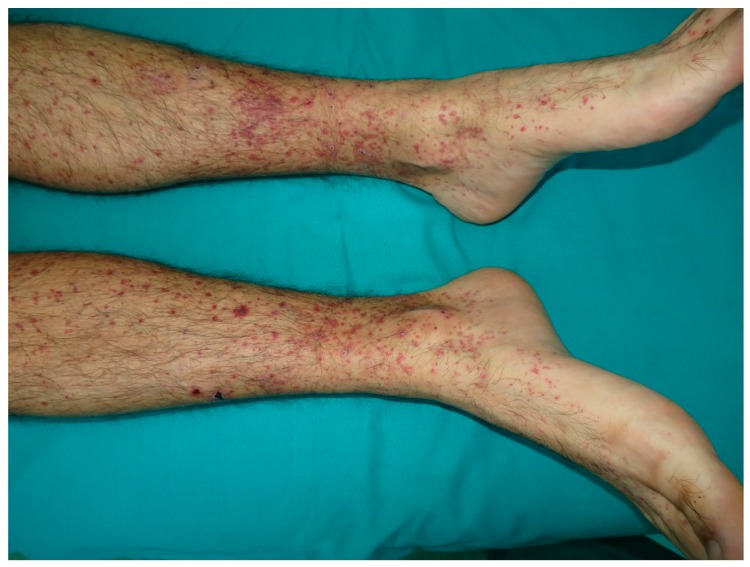
Vasculitis. Palpable purpuric papules on the lower extremities.

Leukocytoclastic vasculitis, also known as “hypersensitivity vasculitis”, is a histopathologic diagnosis given to cutaneous, small vessel vasculitis, characterized by the inflammation of the walls of postcapillary venules [113]. The clinical features of leukocitoclastic vasculitis include palpable purpura, nodules, hemorrhagic vesicles, bullae, and livedo reticularis, mainly distributed in the lower extremities (Figure 8) [114]. Extracutaneous involvement is seen in approximately 30% of patients. Systemic vasculitis shows a predilection for certain organs, such as the kidneys and lungs. In most cases, leucocytoclastic vasculitis is mediated by immunocomplex deposition, with the antigen being either exogenous or endogenous [115,116,117,118].

When leukocytoclastic vasculitis is suspected, a biopsy should be performed, preferably in the first 24 to 48 h of lesion onset. Additionally, direct immunofluorescence should be performed to evaluate for the presence of immunoglobulins. If no systemic symptoms are present, laboratory testing, including a complete blood count, measurement of the erythrocyte sedimentation rate, basic metabolic panel, liver function tests, and urinalysis should be done as well. If there is concern for systemic involvement, more extensive workup can be fulfilled. Around 90% of leukocytoclastic vasculitis cases are self-limited, showing spontaneous resolution within weeks to months. The treatment depends on the severity of the disease and can range from an oral corticosteroid course to various steroid-sparing agents [113,114].

There are sporadic reports about the association between CD and cutaneous vasculitis (CV) [115,116,117,118,119]. The coexistence of these two entities might be related to increased intestinal permeability [120], and immune complexes, originating from exogenous or endogenous antigens, might circulate because of the impaired phagocytic function of the reticular endothelium system and be deposited in the skin. As seen in inflammatory bowel disease (IBD), exogenous antigens may permeate the damaged CD mucous in larger quantities than normal. This is reflected by significant serum milk and gluten fraction antibody titers. Moreover, autoimmune sensitization may result because of the release of endogenous antigens from the damaged small bowel mucosa. Meyers et al. [118] described a case of CV associated with CD and the remission of skin lesions after the treatment with a strict GFD. Treatment with a GFD may improve CV lesions in cases associated with CD [9,10,11].

## 11. Other Skin Conditions Found in Patients with CD

As reported by Humbert et al. and Caproni et al., in addition to skin diseases with proven association with CD and those improved by a GFD and/or with positivity of celiac serological markers, there are also fortuitous associations with other skin conditions [9,10,11]. Some of these associations are more common than others.

Juvenile dermatomyositis and dermatomyositis have been reported in association with CD in adult patients. In particular, when patients are newly diagnosed with these conditions, even in the absence of gastrointestinal symptoms, screening for CD should be performed. Clinical manifestations of dermatomyositis may respond to a GFD [121,122,123,124].

The possible association between CD and vitiligo is controversial. There are few cases that have reported the improvement of vitiligo in patients that started a GFD. A common basic autoimmune mechanism has been hypothesized [125,126].

Many similarities exist between the pathogeneses of CD and SLE, but it is still unknown whether there is a true association or not [127,128,129].

Other reported and less frequent associations include lichen planus and lichen sclerosus [130,131,132,133,134,135,136,137,138], linear IgA bullous dermatosis [139,140], prurigo nodularis [141], pityriasis rubra pilaris and erythroderma [142], erythema elevatum diutinum [143,144,145], necrolytic migratory erythema [146,147,148], pityriasis lichenoides [140], erythema nodosum [140,149,150,151], porphyria [152,153], cutaneous amyloidosis [154], generalized acquired cutis laxa [155,156], acquired hypertrichosis lanuginosa [157], ichthyosis [158], partial lipodystrophy [159], transverse leukonychia [160], atypical mole syndrome, and congenital giant nevus [161]. Finally, we want to mention that earlier studies reported an increased risk of malignant melanoma in patients with CD, but a recent study refuted this relation [162].

## 12. Other Oral Cavity Disorders

Other oral cavity manifestations among patients with CD have also been described [18,97,98,99,110,163,164]. Rashid et al. described oral and dental manifestations of CD, consisting of enamel defects, delayed eruption, recurrent aphthous ulcers, cheilitis, and atrophic glossitis [96]. Bramanti et al. found atrophic glossitis, angular cheilitis, and burning tongue to be more frequent in CD patients than in control patients [165].

## 13. Conclusions

CD is a systemic process of autoimmune nature that affects genetically predisposed people in relation to a permanent intolerance to gluten and related proteins. It has a worldwide distribution, a slight female predominance, and can appear at any age, with a variable clinical course, ranging from subclinical or asymptomatic cases to very severe ones. In the physical examination of these patients, it is very important to recognize the presence of several types of dermatitis which are found in association with CD, such as urticaria (HR: 1.51, CI: 1.36–1.68), chronic urticaria (HR: 1.92, CI: 1.48–2.48), atopic dermatitis (OR: 3.17, CI: 1.02–9.82), psoriasis (HR: 1.72, CI = 1.54–1.92), aphthous stomatitis (OR: 3.79, CI = 2.67–5.39) and rosacea (HR: 1.46, CI = 1.11–1.93), and other skin affectation processes that are not so clearly related to CD. All of these diseases may occur in the form of outbreaks, accompanied generally by pruritus, which negatively affects their quality of life. Their relationship with gluten is through allergic, inflammatory, immunological, and mixed processes. The recognition of their probable relationship facilitates the diagnosis of CD, and the establishment of a GFD improves the evolution of cutaneous lesions and in some cases, full recovery is achieved.

It is very important to emphasize that the classic presentations of CD with associated malabsorption syndrome are currently considered to be exceptional, especially from the age of two years, and the predominant forms are those with mild, fluctuating, or even absent digestive symptoms and a wide range of extra-intestinal manifestations [166,167,168,169,170]. Many undiagnosed celiac patients underestimate their multiple and frequent discomfort from digestive and more general causes because they have grown accustomed to living with a state of chronic poor health as though it is normal. They are only able to recognize that they really did have symptoms related to the consumption of gluten when they start the GFD and the improvement becomes obvious [171,172].

## Figures and Tables

**Table 1:** Strength of evidence for the association between celiac disease and skin diseases.

Type of Mechanism	Diseases Found in Association with Celiac Disease	Relative Risk in Celiac Disease Compared to the General or Control Population [Reference]	Fortuitous Association (Sporadic Cases)
**Allergic**	Urticaria Chronic urticaria Atopic dermatitis	HR: 1.51 (CI = 1.36–1.68) [12] HR: 1.92 (CI = 1.48–2.48) [12] OR:3.17 (CI = 1.02–9.82) [13]	Prurigo nodularis
**Inflammatory**			Pityriasis rubra pilaris Erythroderma Erythema elevatum diutinum Necrolytic migratory erythema Pityriasis lichenoides Erythema nodosum
**Immune-mediated**	Psoriasis	HR: 1.72 (CI = 1.54–1.92) [14] OR: 1.44 (CI = 1.40–1.92) [15] OR: 3.09 (CI = 1.92–4.97) [16] IgA anti-gliadin: OR: 2.36 (CI = 1.15–4.83) [17]	
**Autoimmune**			Alopecia areata Cutaneous vasculitis Ig A linear dermatosis Dermatomyositis Vitiligo Lupus erythematous Lichen sclerosus
**Miscellaneous**	Aphthous stomatitis Rosacea	OR: 3.79 (CI = 2.67–5.39) [18] HR: 1.46 (CI = 1.11–1.93) [19]	Cutaneous amyloidosis Annular erythema Partial lipodystrophy Generalized acquired cutis laxa Icthyosis Transverse leukonychia Porphyria Hypertricosis lanuginosa

GFD: gluten-free diet; CD: celiac disease; HR: hazard ratio; OR: odds ratio; CI: 95% confidence interval; IgA: immunoglobulin A.
